# Detection of SARS-CoV-2 RNA in a Zoo-Kept Red Fox (*Vulpes vulpes*)

**DOI:** 10.3390/v16040521

**Published:** 2024-03-28

**Authors:** Tatjana Chan, Julia Ginders, Evelyn Kuhlmeier, Marina L. Meli, Eva Bönzli, Theres Meili, Julia Hüttl, Jean-Michel Hatt, Karin Hindenlang Clerc, Anja Kipar, Fabia Wyss, Christian Wenker, Marie-Pierre Ryser-Degiorgis, Cecilia Valenzuela Agüí, Christian Urban, Christian Beisel, Tanja Stadler, Regina Hofmann-Lehmann

**Affiliations:** 1 Clinical Laboratory, Department of Clinical Diagnostics and Services, and Center for Clinical Studies, Vetsuisse Faculty, University of Zurich, Winterthurerstrasse 260, 8057 Zurich, Switzerland; tatjana.chan@uzh.ch (T.C.); julia.ginders@uzh.ch (J.G.); ekuhlmeier@vetclinics.uzh.ch (E.K.); mmeli@vetclinics.uzh.ch (M.L.M.); julia.huettl@uzh.ch (J.H.); 2 Clinic for Zoo Animals, Exotic Pets and Wildlife, Department of Small Animals, Vetsuisse Faculty, University of Zurich, Winterthurerstrasse 260, 8057 Zurich, Switzerland; jmhatt@vetclinics.uzh.ch; 3 Foundation Wildnispark Zurich, Alte Sihltalstrasse 38, 8135 Sihlwald, Switzerland; karin.hindenlang@wildnispark.ch; 4 Institute of Veterinary Pathology, Vetsuisse Faculty, University of Zurich, Winterthurerstrasse 268, 8057 Zurich, Switzerland; anja.kipar@uzh.ch; 5 Zoologischer Garten Basel AG, Binningerstrasse 40, 4054 Basel, Switzerland; fabia.wyss@zoobasel.ch (F.W.); christian.wenker@zoobasel.ch (C.W.); 6 Institute for Fish and Wildlife Health, Vetsuisse Faculty, University of Bern, 3012 Bern, Switzerland; marie-pierre.ryser@unibe.ch; 7 Department of Biosystems Science and Engineering, ETH Zurich, Schanzenstrasse 44, Postfach, 4009 Basel, Switzerland; cecilia.valenzuela@bsse.ethz.ch (C.V.A.); christian.beisel@bsse.ethz.ch (C.B.); tanja.stadler@bsse.ethz.ch (T.S.); 8 SIB Swiss Institute of Bioinformatics, 1015 Lausanne, Switzerland; 9 Functional Genomics Center, ETH Zurich and University of Zurich, Winterthurerstrasse 190, 8057 Zurich, Switzerland; christian.urban@uzh.ch

**Keywords:** One Health, wildlife, wild animal species, zoo animals, COVID-19, red fox

## Abstract

Many different animal species are susceptible to SARS-CoV-2, including a few Canidae (domestic dog and raccoon dog). So far, only experimental evidence is available concerning SARS-CoV-2 infections in red foxes (*Vulpes vulpes*). This is the first report of SARS-CoV-2 RNA detection in a sample from a red fox. The RT-qPCR-positive fox was zoo-kept together with another fox and two bears in the Swiss Canton of Zurich. Combined material from a conjunctival and nasal swab collected for canine distemper virus diagnostics tested positive for SARS-CoV-2 RNA with Ct values of 36.9 (E gene assay) and 35.7 (RdRp gene assay). The sample was analysed for SARS-CoV-2 within a research project testing residual routine diagnostic samples from different animal species submitted between spring 2020 and December 2022 to improve knowledge on SARS-CoV-2 infections within different animal species and investigate their potential role in a One Health context. Within this project, 246 samples from 153 different animals from Swiss zoos and other wild animal species all tested SARS-CoV-2 RT-qPCR and/or serologically negative so far, except for the reported fox. The source of SARS-CoV-2 in the fox is unknown. The fox disappeared within the naturally structured enclosure, and the cadaver was not found. No further control measures were undertaken.

## 1. Introduction

Many different mammalian species are susceptible to infection with SARS-CoV-2, the virus that led to the COVID-19 pandemic which began in early 2020. Depending on the source, up to 904 events in 34 animal species from 40 countries worldwide were reported by January 2024 [1,2]. Susceptible animal species also include members of the canid family, the domestic dog (*Canis lupus familiaris*) and the racoon dog (*Nyctereutes procyonoides*). At least in the domestic dog, the susceptibility to SARS-CoV-2 infection is lower than, e.g., in humans, mustelids and felids, and experimentally challenged coyotes (*Canis latrans*) did not become infected [3]. So far, natural SARS-CoV-2 infection has not been reported in red foxes [1,4]. However, juvenile red foxes (*Vulpes vulpes*) were susceptible to experimental SARS-CoV-2 infection, using the instillation of the cell-cultured virus into their nares [3]. All six animals in the experimental study shed infectious virus orally and nasally starting one day after inoculation; shedding persisted for two to three days and ceased by day 5 [3]. No gross lesions were observed upon post mortem examination in these animals. A study undertaken on 204 red foxes sampled in Croatia between June 2020 and March 2021 did not find evidence of SARS-CoV-2 infection; faecal and muscle samples all tested negative by RT-qPCR, and although some reactivity was found from muscle extracts in a commercial multispecies SARS-CoV-2 antibody enzyme-linked immunosorbent assay (ELISA), all samples tested negative in a subsequent surrogate virus neutralisation assay (sVNT) [5]. The authors concluded that spillover from humans to foxes had not occurred. Furthermore, an earlier study on 89 free-living foxes in China did not find any seropositives [6]. Thus, the present report is the first to demonstrate viral RNA after natural SARS-CoV-2 exposure in a wild carnivore. The red fox was zoo-kept; the source of the virus is unknown.

## 2. Materials and Methods

### 2.1. Animals and Samples

Materials included in this retrospective study were residual samples taken for unrelated routine diagnostic purposes (Table A1 and Table A2) or were collected within a health surveillance programme (Table A3). No extra volume or extra samples were collected for this study. We included in this study all samples from wild animal species, independent of their origin: zoo, privately kept animals or free-ranging animals. The study included a total of 246 samples from 153 animals.

The 174 diagnostic samples had been submitted to the clinical laboratory and were intended for disposal after analysis. They had been collected from 122 animals in Switzerland between February 2020 and December 2022. This included 96 oropharyngeal, nasal, conjunctival and faecal swabs, as well as 78 serum samples. They were used for SARS-CoV-2 RT-qPCR and the detection of antibodies, respectively (Table A1 and Table A2). The material for molecular analysis included samples from four zoo-kept foxes (Table A1; designated Fox 1 to Fox 4) of which one animal (Fox 2) underwent post mortem examination for unrelated reasons. A formalin-fixed and paraffin-embedded lung sample from Fox 2 was subjected to immunohistology for the in situ detection of SARS-CoV-2 nucleoprotein, following a published protocol [7].

In addition, we received 72 samples collected from 31 animals at Zoo Basel, Switzerland (Table A3). Serum, faecal and oropharyngeal samples had been collected in the zoo’s framework of their health surveillance programme between April 2020 and March 2022. These included primarily samples from primates, felids, canids and mustelids. Faecal and oropharyngeal samples (*n* = 51) underwent RT-qPCR; serum samples (*n* = 21) were used for antibody detection against SARS-CoV-2 (Table A3).

### 2.2. Serology

Serum samples were analysed using a commercially available SARS-CoV-2 Surrogate Virus Neutralisation Test Kit (sVNT; GenScript Inc., Piscataway, NJ, USA). The test was performed according to the manufacturer’s instructions and as previously described [8]. Four samples showing sVNT results slightly >20% (cut-off for human samples) were further tested using an in-house-developed ELISA that detects antibodies binding to the SARS-CoV-2 spike glycoprotein receptor-binding domain (RBD), as described in [9] and a multispecies conjugate (SBVMILK; obtained from IDVet ID Screen Schmallenberg Virus Milk Indirect ELISA; Labgene Scientific, Chatel-St-Denis, Switzerland) [10].

### 2.3. Nucleic Acid Extraction and Molecular Assays

All laboratory work involving materials for molecular analysis was performed under laminar flow hoods in designated laboratories for each sample preparation step (extraction, pipetting, PCR, etc.), and the laboratory personnel wore FFP3 masks. Dry oropharyngeal, nasal, conjunctival and faecal swabs submitted for routine diagnostic purposes were resuspended in 400 μL Hank’s Balanced Salt Solution (HBSS) and then incubated at 42 °C for 30 min in a shaking incubator at 600 rpm. Dry oropharyngeal swabs and faecal samples collected at Zoo Basel were put in 1.5 mL screw-lid tubes (Sarstedt AG and Co. KG, Nümbrecht, Germany) prefilled with 300 µL of DNA/RNA shield solution (Zymo Research Europe GmbH, Freiburg, Germany). They were transported in sealable containers (triple packed for biosafety reasons), unpacked in a laminar flow cabinet, and rinsed with 70% ethanol, wiped and stored at −20 °C until further processing. Subsequently, all tubes with cotton swabs were centrifuged at 8000 rpm for one minute. Cotton swabs were inverted within the tubes using tweezers, which were cleaned between each sample with RNAse Away (Thermo Fisher Scientific, Basel, Switzerland) and then with 70% ethanol. The tubes underwent another centrifugation at 8000 rpm for one minute. Using the cleaned tweezers as described above, the cotton swabs were then removed. Samples were stored at −20 °C until further analysis.

TNA were extracted from 200 µL of liquid samples using either a MagNA Pure 96 instrument and the MagNA Pure 96 DNA and Viral NA Small Volume Kit or a MagNa Pure LC2 instrument and the MagNA Pure LC Total Nucleic Acid High Performance Kit (Roche Diagnostics AG, Rotkreuz, Switzerland), according to the manufacturer’s instructions. For each batch of extractions, a negative control (phosphate-buffered saline (PBS) without Ca^2+^ and Mg^2+^, Life Technologies Ltd., Paisley, UK) was included to monitor for potential cross contamination. RNA from fresh and deparaffinized tissue samples was extracted using the RNeasy Mini Kit and the RNeasy FFPE kit (Qiagen, Hilden, Germany), respectively, according to the manufacturer’s instructions.

SARS-CoV-2 RT-qPCR was run on an ABI PRISM 7500 Fast Sequence Detection System (Applied Biosystems, Foster City, CA, USA). Two assays were used to amplify a target in the envelope gene (E) and the RNA-dependent RNA polymerase sequence (RdRp) as previously described [8]. Negative RT-qPCR controls (RNAse-DNase-free water, AppliChem, Darmstadt, Germany), a negative extraction control (PBS) and a positive RT-qPCR control (in vitro transcribed RNA control containing three concatenated sequences of RdRp, E, and nucleocapsid (N) SARS-CoV-2 genes: RNA_Wuhan_RdRp-E-N) were assayed with every run.

Extracted TNA/RNA from the RT-qPCR-positive swab samples were sent for next-generation sequencing to the Genomics Facility Basel, Eidgenössische Technische Hochschule (ETH) Zürich, Basel, Switzerland. Library preparation and whole-genome sequencing was performed on Illumina MiSeq and NovaSeq 6000 NGS systems as previously described [11], following the ARTIC v3 protocol. Due to the low viral concentration in the sample, another sequencing attempt was made at the Functional Genomics Center Zurich (FGCZ) with a total RNA approach to maximize the probability of recovering a genetic sequence. Library preparation was performed with the SMARTer Stranded Total RNA-Seq Kit v3 - Pico Input Mammalian, followed by an enrichment step for SARS-CoV-2 with myBaits Hybridization capture kit, and 100 bp single end sequencing was performed on Illumina NextSeq2000. For the quality control and processing of raw sequencing reads, the bioinformatic pipeline V-pipe [12] was used, with the SARS-CoV-2 base configuration. Sequences with <20,000 bases called were rejected.

## 3. Results

A clinically healthy red fox (Fox 1) tested SARS-CoV-2 RT-qPCR positive. The animal lived in a zoo in the Canton of Zurich, in an enclosure with a second fox (Fox 2) and two bears. Fox 1 was quarantined on 25 March 2022 because two other adult foxes, Fox 3 and Fox 4, living in another enclosure at the same zoo, had tested positive for canine distemper virus (CDV). The two foxes (Fox 3 and Fox 4) had been presented (on 11 March 2022) to the University Animal Hospital in Zurich (Clinic for Zoo Animals, Exotic Pets and Wildlife) and a pooled conjunctival swab sample was positive for CDV RNA by RT-qPCR. Both foxes were euthanized because of the distemper diagnosis. The pooled conjunctival swab sample of Fox 3 and Fox 4 tested negative for SARS-CoV-2 RNA.

Fox 1 was vaccinated against CDV and sampled on 03/29/2022. Combined material from the conjunctival and nasal swabs tested negative for CDV RNA. However, the sample tested positive for SARS-CoV-2 RNA with Ct values of 36.9 (E gene assay) and 35.7 (RdRp gene assay). The limited remaining nucleic acid material was subjected to next-generation sequencing attempts; however, no sequence could be retrieved. Fox 1 was subsequently placed in the enclosure where the two adult foxes (Fox 3 and Fox 4) that had been euthanized had lived formerly. Fox 1 disappeared within the naturally structured enclosure; a cadaver was not found, and hence, no further samples were available.

Fox 2, a one-year-old male fox that lived in the bear enclosure together with Fox 1, was found dead on 22 March 2022, 7 days before Fox 1 tested SARS-CoV-2 RT-PCR positive. Fox 2 underwent a full post mortem examination at the Institute of Veterinary Pathology and was diagnosed with haemothorax, acute mediastinal haemorrhages, skin lacerations at the forelimbs and subluxation of the dorsal joints of the thoracic ribs, all consistent with trauma (compression of the ribcage, bruises of the forelimbs), likely due to biting by a bear, plus severe verminous pneumonia (due to infection with the nematode *Angiostrongylus vasorum*). Tissue samples taken from the nose and the third eyelid from this fox tested negative by RT-qPCR for CDV and SARS-CoV-2 RNA. The lung tested negative by immunohistochemistry for SARS-CoV-2 nucleoprotein, and yielded an inconclusive SARS-CoV-2 RT-qPCR due to insufficient TNA quality (eukaryotic 18s ribosomal RNA control RT-PCR (Thermo Fisher) negative/low positive) after RNA extraction from deparaffinised lung sections.

Apart from the combined conjunctival and nasal swabs from Fox 1, all samples tested within this study were SARS-CoV-2 negative in RT-qPCR (E and RdRP Ct values 45; Table A1 and Table A3). They originated from 74 different animals, including 16 free-ranging lynxes and 12 zoo-kept large felids of five different species, 16 Mustelidae (ferret, badger, meerkat, and dwarf mongoose), a free-ranging wolf and a zoo-kept wild dog as well as six Hominidae.

Moreover, among the 99 available serum samples (Table A2 and Table A3), all but four tested negative in sVNT using the cut-off for human samples, which was 20% according to the manufacturer at the time. Only one of the diagnostic samples from a mongoose (Table A2) and two meerkats and a cotton-top tamarin from Zoo Basel (Table A3) had sVNT values slightly above 20% (22.1%, 22.8%, 21.3%, and 20.9%). No cut-offs were available for these species, but for other animal species, cut-offs had been determined to be higher than those for humans using this assay [9]. Moreover, the four samples were tested using an ELISA and a multispecies conjugate, as described in [10]. All four samples tested negative for SARS-CoV-2 binding antibodies. Therefore, all 99 available serum samples, including in our study, were judged seronegative.

## 4. Discussion

This is the first report of a SARS-CoV-2 RNA-positive fox after presumptive natural exposure to the virus. Besides the domestic dog and the racoon dog, the red fox is the third canid species shown to be RT-PCR positive for SARS-CoV-2. Wild canids have not been a primary focus of research, since they are presumably less susceptible to SARS-CoV-2 infection than, e.g., felids or mustelids. However, red foxes live near human settlements and municipal waste and might therefore be at risk of SARS-CoV-2 infections by spillover from infected humans.

The source of SARS-CoV-2 in the fox is unknown. None of the animal caretakers in contact with the fox had knowingly been infected with SARS-CoV-2 at the time. However, due to the retrospective nature of the current study, the targeted testing of the caretakes was not an option, and no human samples were available. In other zoo-kept animals, infected caretakers were primarily suspected as a source of SARS-CoV-2 infection [13,14,15]. Possible sources of the virus in the current case other than the animal caretakers include whole-body feeding of dead food animals (rats, rabbits) or other animals, such as free-living rodents, martens or even foxes. Transmission of SARS-CoV-2 in animals including cross-species transmission among different species has been recently reviewed [16,17]. SARS-CoV-2 remains viable in contaminated food (animals) for days to weeks at room temperature, 4° as well as frozen at −20° [18], and contaminated water was suspected as a source of infection in wild river otters and minks [19,20].

In the experimentally infected juvenile red foxes, oral swabs from the six animals were SARS-CoV-2 RT-qPCR positive at day 2 [3]. Shedding seemed short-lived; virus isolation was only positive up to day 3 and not anymore at day 5, while viral RNA detected by RT-qPCR was found until day 7 but not anymore at day 14. Whether the shedding pattern after experimental challenge mirrors the natural situation is unknown. Assuming this is the case, the probability to detect SARS-CoV-2 viral RNA shedding in a naturally infected fox is low. Nonetheless, considering the experimental study, one could assume that Fox 1 reported herein was exposed to SARS-CoV-2 a few days before sampling. Ct values were rather high corresponding to low SARS-CoV-2 loads in the sample. However, the viral RNA loads in Fox 1 are difficult to compare with the loads in the experimentally infected foxes, not only because of the different nature of infection but also due to potential differences in the methodological approach.

In experimentally infected foxes, seroconversion and a neutralizing response started by day 7 in all three tested animals [3]. Apart from virus isolation after experimental infection from oropharyngeal and nasal samples, seroconversion proves the presence of an active infection after experimental challenge. In the current case, no further samples and, thus, no serum samples were available to corroborate an active infection of the fox. We cannot completely rule out potential contamination of the fox sample during collection or analysis. However, every possible precaution was taken in the laboratory to avoid potential contamination.

Infectious virus was isolated after experimental infection upon necropsy of the foxes from nasal concha but not from any other tissue [3]. Fox 2, who was living in the enclosure with the SARS-CoV-2-positive Fox 1 in the current study, underwent a full post mortem examination. Samples from the nose and the third eyelid were available for RT-qPCR, and both tested negative. The lungs tested negative by immunohistochemistry for SARS-CoV-2 nucleoprotein. Our results seem thus inconclusive concerning a potential SARS-CoV-2 infection in Fox 2 as no material from the nasal concha was collected during the necropsy. Fox 2 was most likely bitten to death by a bear but had also suffered from verminous pneumonia.

We are aware of the fact that the set-up of the study does not allow for any general statements concerning the prevalence of SARS-CoV-2 infections in the different animal species under investigation. We used convenience samples that were available to us from “interesting” species not examined so often. In case of a positive result, like for the fox, such studies help to recognize additional species that may be susceptible to natural SARS-CoV-2 infections, and in the worst case, may provide a possible new reservoir for the virus. In addition, our report of SARS-CoV-2-negative data may contribute to future meta-data analysis studies that further the knowledge in the field.

## 5. Conclusions

The herein-reported fox is the first SARS-CoV-2 RNA-positive fox documented in Switzerland. To the best of our knowledge, it is also the first to provide evidence of a natural SARS-CoV-2 infection in a fox. SARS-CoV-2 infection in the red fox seems rare; none of the other studies investigating foxes reported any positive animals so far [5,6]. A serological survey study on red foxes in Switzerland is ongoing. Since all four foxes from this zoo were either euthanized, died or, in one case, disappeared, no further control measures were deemed necessary.

## Data Availability

All data are presented within this manuscript. The RT-qPCR confirmed fox (Fox 1) was reported to the WOAH [21] and to ProMed [22].

## Appendix A

**Table A1:** Overview of residual samples from wild animal species submitted for routine diagnostic purposes, which were tested for the presence of SARS-CoV-2 RNA.

Species	Number of Animals	Origin	Number of Oropharyngeal/Faecal Samples	Results SARS-CoV-2 RT-qPCR
**Canidae**				
Red fox (*Vulpes vulpes*)	4 ^1^	Zoo	5	Positive (1/4)
Wolf (*Canis lupus*)	1	Free-ranging	2	Negative
**Felidae**				
Lynx (*Lynx lynx*) ^2^	16	Free-ranging	40	Negative
Cheetah (*Acinonyx jubatus*)	1	Zoo	1	Negative
Snow leopard (*Panthera uncia*)	5	Zoo	12	Negative
Lion (*Panthera leo*)	1	Zoo	1	Negative
Tiger (*Panthera tigris*)	2	Zoo	6	Negative
**Mustelidae**				
Ferret (*Mustela furo*)	6	Privately owned	15	Negative
Badger (*Meles meles*)	1	Free-ranging	1	Negative
**Hominidae**				
Gorilla (*Gorilla gorilla*)	1	Zoo	1	Negative
**Callitrichidae**				
Marmoset (*Callithrix jacchus*)	2	Zoo	4	Negative
**Sciuridae**				
Asiatic striped squirrel (*Tamiops swinhoei*)	2	Zoo	4	Negative
**Phocidae**				
Harbor Seal (*Phoca vitulina*)	1	Zoo	3	Negative
**Erinaceidae**				
Hedgehog (*Erinaceus europaeus*)	1	Free-ranging	1	Negative
Total	44		96	

^1^ Foxes designated Fox 1 to Fox 4; combined material from a conjunctival and a nasal swab from Fox 1 tested SARS-CoV-2 RT-qPCR positive. ^2^ Lynx samples included in this study had been collected between February 2020 and May 2021.

**Table A2:** Overview of residual samples from wild animal species submitted for routine diagnostic purposes, which were tested serologically for SARS-CoV-2 antibodies.

Species	Origin	Number of Serum Samples	Result Serology ^2^
**Felidae**			
Lynx (*Lynx lynx*) ^1^	Free-ranging	2	Negative
**Mustelidae**			
Asian small-clawed otter (*Aonyx cinereus)*	Zoo	1	Negative
**Leporidae**			
European rabbit *(Oryctolagus cuniculus domesticus)*	Privately owned	12	Negative
**Camelidae**			
Alpaca	Privately owned	16	Negative
Camel (*Camelus ferus*)	Zoo/privately owned	2	Negative
Dromedary (*Camelus dromedarius*)	Privately owned	4	Negative
Llama (*Lama glama*)	Zoo	1	Negative
**Bovidae**			
Arabian Oryx (*Oryx leucoryx*)	Zoo	1	Negative
Impala (*Aepyceros melampus*)	Zoo	1	
Alpine ibex (*Capra ibex*)	Zoo	3	Negative
**Cervidae**			
Reindeer (*Rangifer tarandus*)	Privately owned	1	Negative
**Chinchillidae**			
Chinchilla (*Chinchilla*)	Privately owned	1	Negative
**Elephantidae**			
Asian Elephant (*Elephas maximus*)	Zoo	5	Negative
**Folivora**			
Two-toed sloths (*Choloepus*)	Zoo	1	Negative
**Macropodidae**			
Western grey Kangaroo (*Macropus fuliginosus)*	Zoo	2	Negative
**Herpestidae**			
Yellow mongoose (*Cynictis penicillata*)	Zoo	1	Negative
**Cavidae**			
Guinea pig (*Cavinae*)	Privately owned	8	Negative
**Otaridae**			
Eared seal (*Zalophus californianus*)	Zoo	2	Negative
**Agamidae**			
Bearded dragon (*Pogona vitticeps*)	Privately owned	1	Negative
**Procaviidae**			
Rock hyrax (*Procavia capensis*)	Zoo	2	Negative
**Primates**			
Chimpanzee (*Pan troglodytes*)	Zoo	5	Negative
Orangutan	Zoo	1	Negative
Geoffroy’s spider monkey (*Ateles geoffroyi*)	Zoo	1	Negative
Dschelada (*Theropithecus gelada*)	Zoo	1	Negative
**Lemuridea**			
White-belted ruffed lemur (*Varecia variegata subcincta*)	Zoo	2	Negative
Red ruffed lemur (*Varecia rubra*)	Zoo	1	Negative
**Total**		**78**	**Negative**

^1^ Lynx samples included in this study had been collected in October and November 2020. ^2^ sVNT in all but one sample < 20% (cut-off for human samples); only the mongoose sample was slightly above 20% (22.1%); this was judged as negative since no cut-offs were available for this species, but for other animal species, cut-offs had been determined to be higher than for humans using this assay [23]. Moreover, the sample tested negative in the RBD ELISA.

**Table A3:** Overview of samples taken from zoo animals at Zoo Basel for health surveillance and analysed for SARS-CoV-2 RNA and antibodies to SARS-CoV-2.

Species	Number of Animals	Number of Faecal/Oropharyngeal Samples	Results SARS-CoV-2 RT-qPCR	Number of Serum Samples	Result Serology ^1^
**Canidae**					
African wild dog (*Lycaon pictus*)	1	1	Negative	-	-
**Felidae**					
Cheetah (*Acinonyx jubatus*)	1	2	Negative	1	Negative
Lion (*Panthera leo*)	2	4	Negative	1	Negative
**Mustelidae**					
Meerkat (*Suricata suricatta)*	5	8	Negative	5	Negative
Dwarf mongoose (*Helogale parvula*)	4	8	Negative	-	-
**Otaridae**					
California sea lion (*Zalophus californianus*)	2	2	Negative	2	Negative
**Hominidae**					
Sumatran orangutan (*Pongo abelii*)	2	4	Negative	2	Negative
Western lowland gorilla (*Gorilla gorilla gorilla*)	1	2	Negative	-	-
Western chimpanzee (*Pan troglodytes verus*)	2	2	Negative	1	Negative
**Atelidae**					
Brown woolly monkey (*Lagothrix lagothricha*)	2	3	Negative	-	-
Black-handed spider monkey (*Ateles geoffroyi*)	2	3	Negative	1	Negative
**Pitheciidae**					
Coppery titi (*Plecturocebus cupreus*)	1	2	Negative	1	Negative
**Callitrichidae**					
Cotton-top tamarin (*Saguinus oedipus*)	1	2	Negative	1	Negative
Golden lion tamarin (*Leonthopithecus rosalia*)	1	2	Negative	2	Negative
**Cebidae**					
Bolivian squirrel monkey (*Saimiri boliviensis boliviensis*)	1	2	Negative	1	Negative
**Lemuridae**					
White-belted ruffed lemur (*Varecia variegata subcincta*)	3	4	Negative	3	Negative
**Total**	**31**	**51**		**21**	

^1^ sVNT in most samples < 20% (cut-off for human samples); only two meerkats and a cotton-top tamarin were slightly above 20% (22.8%, 21.3%, 20.9%); this was judged as negative since no cut-offs were available for these species, but for other animal species, cut-offs had been determined to be higher than for humans using this assay [23]. Moreover, all three samples tested negative in the RBD ELISA.
